# PKA Inhibitor H89 (N-[2-p-bromocinnamylamino-ethyl]-5-isoquinolinesulfonamide) Attenuates Synaptic Dysfunction and Neuronal Cell Death following Ischemic Injury

**DOI:** 10.1155/2015/374520

**Published:** 2015-09-13

**Authors:** Juhyun Song, So Yeong Cheon, Won Taek Lee, Kyung Ah Park, Jong Eun Lee

**Affiliations:** ^1^Department of Anatomy, Yonsei University College of Medicine, Seoul 120-752, Republic of Korea; ^2^BK21 Plus Project for Medical Sciences and Brain Research Institute, Yonsei University College of Medicine, Seoul 120-752, Republic of Korea

## Abstract

The cyclic AMP-dependent protein kinase (PKA), which activates prosurvival signaling proteins, has been implicated in the expression of long-term potentiation and hippocampal long-term memory. It has come to light that H89 commonly known as the PKA inhibitor have diverse roles in the nervous system that are unrelated to its role as a PKA inhibitor. We have investigated the role of H89 in ischemic and reperfusion injury. First, we examined the expression of postsynaptic density protein 95 (PSD95), microtubule-associated protein 2 (MAP2), and synaptophysin in mouse brain after middle cerebral artery occlusion injury. Next, we examined the role of H89 pretreatment on the expression of brain-derived neurotrophic factor (BDNF), PSD95, MAP2, and the apoptosis regulators Bcl2 and cleaved caspase-3 in cultured neuroblastoma cells exposed to hypoxia and reperfusion injury. In addition, we investigated the alteration of AKT activation in H89 pretreated neuroblastoma cells under hypoxia and reperfusion injury. The data suggest that H89 may contribute to brain recovery after ischemic stroke by regulating neuronal death and proteins related to synaptic plasticity.

## 1. Introduction 

Protein kinase A (PKA) [1] acts to phosphorylate other proteins, regulating them in a reversible manner. When cyclic adenosine monophosphate (cAMP) binds to the subunits of PKA, they undergo a conformational change that promotes phosphorylation [2]. PKA is implicated also in neural health. It stimulates neurite outgrowth in neurons and neuronal cell lines [3, 4] and promotes axon regeneration in vivo [5, 6]. cAMP/PKA signaling affects long-term synaptic plasticity and long-term memory [7].

Many studies that evaluate the role of PKA, which include smooth muscle cells [8, 9], neuronal tissue [10, 11], and epithelial cells [12, 13], have relied on the isoquinoline derivative N-[2-p-bromocinnamylamino-ethyl]-5-isoquinolinesulfonamide (H89), an inhibitor of PKA. H89 has an inhibition constant (*K*_*i*_) of 0.05 mM in its inhibition of PKA [14, 15]. However, effects of H89 that are unrelated to its inhibition have been observed. In a kinase study, at a concentration of 10 *μ*M, H89 inhibited the activity of the protein kinases Rho-associated kinase- (ROCK-) II, MSK1 and the ribosomal protein S6 kinase *β*-1 (S6K1) far more potently than it inhibited PKA itself [16]. In addition, H89 10 *μ*M maintains the neurite outgrowth of neuroblastoma cells [17]. There are several reports that H89 reduced Ca^2+^ uptake into the sarcoplasmic reticulum by attenuating the Ca^2+^-ATPase's [18] affinity for calcium [19]. At 20 *μ*M, H89 prevented the glucose-induced increase in cytosolic calcium in pancreatic islets and attenuated the release of calcium in a differentiated *β*-cell line. In a study of expression of myelin basic protein in oligodendrocytes, H89 is involved in the phosphorylation of extracellular-signal–regulated kinase 1 and 2 (ERK 1 and 2) phosphorylation in response to insulin-like growth factor-1 [20] and it lowered potassium current through voltage-gated channels in rat myocytes [21].

Of particular interest is the H89 inhibition of S6K1, noted above. S6K1 is a downstream target of the mammalian target of rapamycin (mTOR) protein, which regulates the autophagy pathway [22] and is a mechanism target for regulation of cell size [23]. Several researchers have questioned the role of PKA in autophagy, since the studies rely at least in part on the selectivity of H89, which they consider uncertain [24, 25]. The second issue involves the action of H89 itself. Clearly, it has physiological effects unrelated to PKA. We have elected to examine those effects and chose to focus on H89's role in neural health, especially ischemic stroke.

Cerebral ischemia leads to neuronal death and synaptic dysfunction, resulting in cognitive decline [26–29]. Understanding the pathogenesis after ischemic stroke should inform medical care and maximize recovery. In the present study, we investigated the role of H89 in many aspects of nervous system function. Specifically, we examined its role in the expression of brain-derived neurotrophic factor (BDNF) in the development of neurites to axons [30–32], learning and memory [33], synaptic plasticity [34], the expression of B-cell lymphoma 2 (Bcl2) [35, 36] as it relates to neuronal death, the expression of synaptophysin [37], postsynaptic density protein 95 (PSD-95) [38, 39] as it relates to synaptic plasticity, and the expression of microtubule-associated protein 2 (MAP2). The latter interacts with actin filaments, shown to be necessary for neurite outgrowth [40–43] in a middle cerebral artery occlusion (MCAO) animal model and in an* in vitro* study. In present study, we suggest that H89 may confer protection from brain damage following cerebral ischemia.

## 2. Materials and Methods

### 2.1. Animal Model

Male C57BL/6 mice (Orient, GyeongGi-Do, Korea) that were eight-to-twelve weeks old were used in this study. Hypoxia followed by reperfusion (H/R) was imposed by subjecting mice to transient focal cerebral ischemia by intraluminal middle cerebral artery blockade with a nylon suture, as previously described [44]. After 60 min of MCAO, blood flow was restored by withdrawing the suture and regional cerebral blood flow was monitored with a laser Doppler flow meter (Transonic Systems, Inc., Ithaca, NY, USA). All animal procedures and experiments were performed in accordance with the Guide to the Care and Use of Laboratory Animals and were approved by the Association for Assessment and Accreditation of Laboratory Animal Care. All procedures were done at room temperature unless indicated otherwise. We used 5 rats in each group for study. Each measurement included 3 repeats per animal.

### 2.2. Immunohistochemistry

Frozen brain sections were cut into 5 *μ*m sections and mounted on clean glass slides (Thermo Scientific, Waltham, MA, USA), air-dried, and fixed in cold acetone for 10 min at −20°C. The slides were washed in Tris-buffered saline (TBS; 20 nM Tris (pH 7.2), 150 mM NaCl), incubated with 0.3% H_2_O_2_ in methanol to quench endogenous peroxidase activity, and washed three times with distilled water, and the sections were blocked with 10% normal rabbit serum. Additional frozen brain sections (20 *μ*m) were fixed in ice-cold acetone for 20 min. To block nonspecific labeling, sections were incubated in 5% bovine serum albumin (BSA; Sigma-Aldrich, St. Louis, MO, USA) in 0.1% phosphate-buffered saline (PBS) for 30 min before addition of primary and secondary antibodies. Primary antibodies for PSD-95 (1 : 100, Millipore, Massachusetts, MA, USA), synaptophysin (1 : 100, Millipore, Massachusetts, MA, USA), and MAP2 (1 : 100, Abcam, Cambridge, MA, USA) were applied to the samples for 24 h at 4°C; then the samples were incubated with the appropriate florescence secondary antibody (1 : 100, Invitrogen, Carlsbad, CA, USA) for 90 min, washed three times for 10 min in PBS with Tween-20 (PBST), and incubated with rhodamine-conjugated sheep anti-rabbit or fluorescein isothiocyanate- (FITC-) conjugated sheep anti-mouse secondary antibody (both diluted to 1 : 200 with 5% BSA fraction V in 0.1% PBST) for 2 h in the dark. This was followed by three washes in PBS and incubation in 1 *μ*g/mL 4′,6-diamidino-2-phenylindole (DAPI; Sigma-Aldrich, St. Louis, MO, USA) for counterstaining. Tissues were then visualized under a confocal microscope (Zeiss LSM 700, Carl Zeiss, Thornwood, NY, USA).

### 2.3. Cell Culture

Neuro2A (N2A) cells purchased from ATCC biotechnology (ATCC, Manassas, VA, USA) were derived from mouse neuroblastoma. The cells exhibited properties of neuronal stem cells and were capable of differentiating into neuron-like cells in the presence of retinoic acid (RA). Undifferentiated N2A cells were cultured in Dulbecco's modified eagle medium (DMEM) supplemented with 10% fetal bovine serum (FBS; Gibco, Grand Island, NY, USA) and 100 *μ*g/mL penicillin-streptomycin (Gibco, Grand Island, NY, USA). N2A cells were passaged at least twice and then plated at 5 × 10^4^ cells/mL in DMEM supplemented with 10% FBS for 24 h, after which the medium was changed to DMEM supplemented with 2% FBS and 20 *μ*M RA for differentiation. Cultures were maintained in a humidified atmosphere of 5% CO_2_ at 37°C. The medium was changed every two days [45].

### 2.4. Hypoxia and Reperfusion (H/R) and H89 Treatment

Confluent cells were transferred to an anaerobic chamber (Forma Scientific, OH, USA, O_2_ tension = 0.1%). They were washed three times with PBS and the culture medium was replaced with deoxygenated, glucose-free balanced salt solution and incubated for 4 h. Following H/R injury, cells were incubated for 18 h under normal growth conditions [46]. H89 (10 *μ*M, Sigma-Aldrich, St. Louis, MO, USA) was treated in the N2A cells at 2 h before H/R injury. In present study, we used the 10 *μ*M concentration of H89, considering previous researches regarding other functions except from PKA inhibitor[17–19, 47, 48].

### 2.5. Neurite Length Measurement

To determine the length of their neurites, the cells were fixed for 20 min in 3.7% formaldehyde. Neurite formation was defined as an outgrowth from the cell body that was longer than the diameter of the cell body. N2A cells in three randomly selected fields (30–100 cells per field) were measured using ImageJ software (ImageJ, Madison, WI, USA) [49]. At least 30 cells per treatment were scored [50].

### 2.6. Reverse Transcription PCR (RT-PCR)

To examine the expression of BDNF, Bcl2, and MAP2 in N2A cells after H/R injury, RT-PCR was performed. Briefly, samples were lysed with TRIzol reagent (Invitrogen, Carlsbad, CA, USA) and total RNA was extracted according to the manufacturer's protocol. Complementary DNA synthesis from mRNA and sample normalization was performed. PCR was performed using the following thermal cycling conditions: 10 min at 95°C, 35 cycles of denaturing at 95°C for 15 sec, annealing for 30 sec at 70°C, elongation at 72°C for 30 sec, final extension for 10 min at 72°C, and maintenance at 4°C. PCR was performed using the following primers (5′ to 3′); BDNF (F): AGT GAT GAC CAT CCT TTT CCT TAC, (R): CCT CAA ATG TGT CAT CCA AGG A, Bcl2 (F): AAG CTG TCA CAG AGG GGC TA, (R): CAG GCT GGA AGG AGA AGA TG, MAP2 (F): TGA AGA ATG GCA GAT GAA C, (R): AGA AGG AGG CAG ATT AGC, GAPDH (F): GGCATGGACTGTGGTCATGAG, (R): TGCACCACCAACTGCTTAGC. PCR products were electrophoresed in 1.5% agarose gels and stained with ethidium bromide.

### 2.7. Western Blot Analysis

After H/R injury, cells were washed rapidly with ice-cold PBS, scraped, and collected. Cell pellets were lysed with ice-cold RIPA buffer (Sigma-Aldrich, St. Louis, MO, USA). The lysates were centrifuged at 13,200 rpm for 1 h at 4°C to produce whole-cell extracts. Protein was quantified with the bicinchoninic acid (BCA) method (Pierce biotechnology, Rockford, IL, USA). Protein (20 *μ*g) was separated on a 10% SDS–polyacrylamide (PAGE) gel and transferred onto a polyvinylidene difluoride (PVDF) membrane. After blocking with 5% BSA (in TBS/Tween [TBS-T]) for 1 h, immunoblots were incubated overnight at 4°C with primary antibodies specific for Bcl2 (1 : 2000, Millipore, Massachusetts, MA, USA), cleaved caspase-3 (1 : 2000, Santa Cruz, Santa Cruz, CA, USA), PSD-95 (1 : 2000, Millipore, Massachusetts, MA, USA), AKT (1 : 2000, Cell signaling, Danvers, MA, USA), p-AKT (1 : 2000, Cell signaling, Danvers, MA, USA), or *β*-actin (1 : 2000, Santa Cruz, Santa Cruz, CA, USA). Next, blots were incubated with horseradish peroxidase- (HRP-) linked anti-mouse and anti-rabbit IgG antibodies purchased from Abcam (Abcam, Cambridge, MA, USA) for 1 h. Enhanced chemiluminescence was performed by electrochemiluminescence (ECL: Pierce Biotechnology, Rockford, IL, USA) [51].

### 2.8. Immunocytochemistry

The expression of BDNF, cleaved caspase-3, Bcl2, and PSD-95 in N2A cells was confirmed by immunocytochemistry. Cells in all experimental groups were washed three times with PBS, fixed with 4% paraformaldehyde for 3 h, and then washed with PBS. N2A cells were permeabilized with 0.025% Triton X-100 and blocked for 1 h with dilution buffer (Invitrogen, Carlsbad, CA, USA). The following primary antibodies: anti-rabbit BDNF (1 : 500, Abcam, Cambridge, MA, USA), anti-rabbit cleaved caspase-3 (1 : 500, Santa Cruz, Santa Cruz, CA, USA), anti-rabbit PSD-95 (1 : 500, Millipore, Massachusetts, MA, USA), anti-mouse Bcl2 (1 : 500, Millipore, Massachusetts, MA, USA) were prepared in dilution buffer, added to samples, and incubated for 3 h. Primary antibody was then removed and cells were washed three times for 3 min each with PBS. Later, samples were incubated with FITC-conjugated goat, anti-rabbit (1 : 200, Jackson Immunoresearch, PA, USA), or rhodamine-conjugated donkey, anti-mouse secondary antibodies (1 : 500, Millipore, Massachusetts, MA, USA) for 2 h. Cells were washed again three times for 3 min each with PBS and stained with 1 *μ*g/mL DAPI (1 : 100, Sigma-Aldrich, St. Louis, MO, USA) for 10 min at room temperature. Fixed samples were imaged using a Zeiss LSM 700 confocal microscope (Carl Zeiss, Thornwood, NY, USA).

### 2.9. Statistical Analysis

Statistical analyses were carried out using SPSS 18.0 software (IBM Corp., Armonk, NY, USA). Data are expressed as mean ± S.E.M. Significant intergroup differences were determined by one-way analysis of variance (ANOVA) followed by Bonferroni post hoc multiple-comparison test. Each experiment included four replicates per treatment. Differences were considered significant at *P* < 0.05 (∗) or *P* < 0.001 (∗∗).

## 3. Results

### 3.1. MCAO Mouse Brain Exhibited Neuronal Death and Synaptic Plasticity Damage

**Figure 1:**
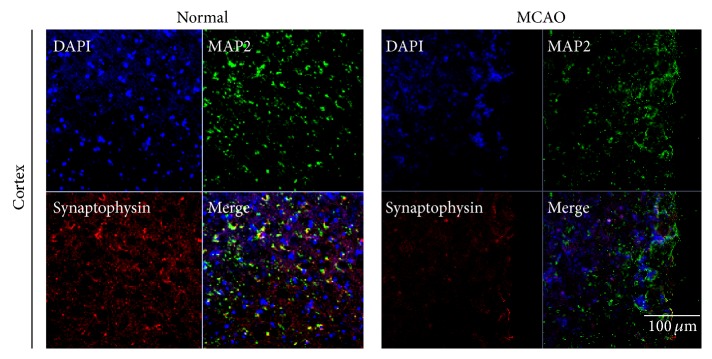
Immunochemical image for confirmation reduced synaptophysin expression in MCAO mouse brain. Immunochemical images showed that synaptophysin-positive cells (red) were decreased as expressed in MCAO mouse cortex. In addition, immunochemical images showed that MAP2- (considered as the neuron specific microtubule protein) positive cells (green) were strongly decreased in MCAO mouse cortex compared to the normal group. We used 5 rats in each groups for study. Each measurement included 3 repeats per animal. Scale bar = 100 *μ*m, synaptophysin: red, MAP2: green, 4′, 6-diamidino-2-phenylindole (DAPI): blue, normal: normal control group, and MCAO: reperfusion 24 hr after MCAO injury.

**Figure 2:**
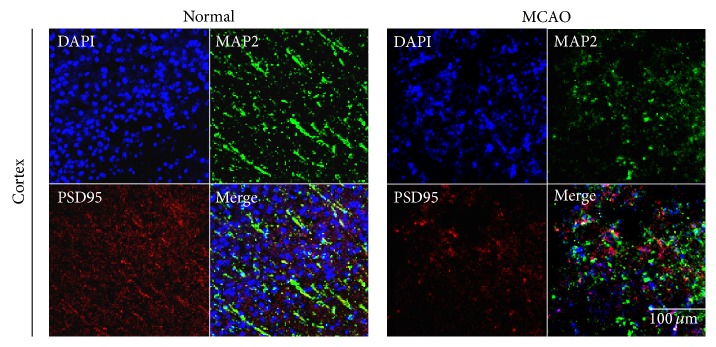
Immunochemical image for confirmation reduced PSD-95 expression in MCAO mouse brain. Immunochemical images showed that PSD-95- (as the post synaptic density protein) positive cells (red) were decreased as expressed in MCAO mouse cortex. Postsynaptic proteins were hardly observed in MCAO mouse brain cortex, whereas the normal cortex was observed evidently. We used 5 rats in each groups for study. Each measurement included 3 repeats per animal. Scale bar = 100 *μ*m, PSD-95: red, MAP2: green, 4′,6-diamidino-2-phenylindole (DAPI): blue, normal: normal control group, and MCAO: reperfusion 24 hr after MCAO injury.

We performed immunohistochemistry of the brain of H/R injured and control mice, using antibodies to synaptophysin (Figure 1), PSD-95 (Figure 2), and MAP2 (Figures 1 and 2). The former two were used as markers of synaptic plasticity; the latter is considered to be a neuronal microtubule protein marker. The immunoreactivity of all three proteins was less in the H/R injured group than in the control group. These results indicate that cerebral ischemia suppresses the expression of synaptophysin, PSD-95, and MAP2 in ischemic brain and that synaptic neuronal microtubule proteins were damaged by ischemic injury.

### 3.2. H/R Injury in Neuro2A Cells Inhibited, and H89 Pretreatment Restored, Neurite Outgrowth

**Figure 3:**
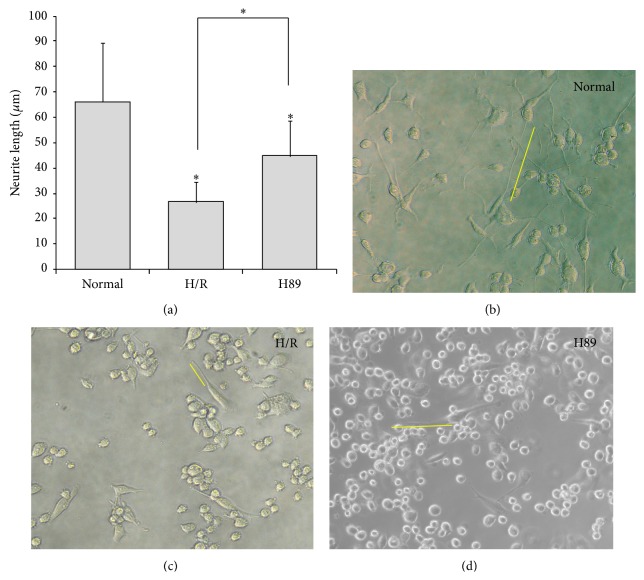
The measurement of neurite outgrowth in Neuro2A cells. (a) The graph of neurite outgrowth length (*μ*m) in all groups. The neurite length significantly decreases in N2A cells against hypoxia reperfusion injury. Data are expressed as mean ± S.E.M. Significant intergroup differences were determined by one-way analysis of variance (ANOVA) followed by Bonferroni post hoc multiple-comparison test. Differences were considered significant at ^∗^ *P* < 0.05. (b) The image using bright field microscope in the normal group shows well developed neurite of N2A cells. (c) The image using bright field microscope in hypoxia reperfusion group shows shorter neurite outgrowth of N2A cells than the normal group. (d) The image using bright field microscope in H89 group shows well developed neurite of N2A cells compared to the hypoxia reperfusion group. Each experiment included 3 repeats per condition. H89 protected N2A cells against the neurite damage under H/R injury. Normal: the normal control group, H/R: 4 hr hypoxia and 18 hr reperfusion injury group, and H89: 2 hr PKA inhibitor H89 treatment group before 4 hr hypoxia and 18 hr reperfusion injury.

Neurite outgrowth of Neuro2A cells was assessed by measuring neurite length with ImageJ software (Figure 3). The average length of normal N2A cells was approximately 65 *μ*m, whereas neurites of cells subjected to H/R injury were approximately 26 *μ*m long (Figure 3(a)). Neurites from cells that had been pretreated with H89 before H/R injury were, on average, approximately 45 *μ*m, or almost twice that of the injured cells that were not pretreated (Figure 3(a)). Bright-field images showed the neurite length in all groups (Figures 3(b), 3(c), and 3(d)). The yellow line in all images permits easy comparison of neurite lengths.

**Figure 4:**
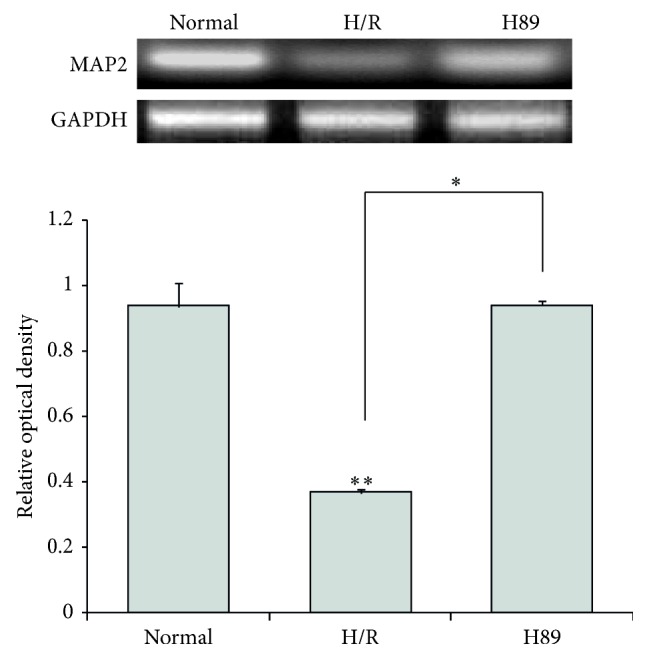
The measurement of MAP2 mRNA level in Neuro2A cells after PKA inhibitor treatment. MAP2 mRNA levels were measured by using RT-PCR. The H89 group showed higher mRNA levels of MAP2 compared to the hypoxia reperfusion injury group. Data were expressed as mean ± S.E.M, and each experiment included 3 repeats per condition. GAPDH was used as a control. Differences were considered significant at ^∗^ *P* < 0.05 and ^∗∗^ *P* < 0.001. Normal: the normal control group, H/R: 4 hr hypoxia and 18 hr reperfusion injury group, and H89: 2 hr PKA inhibitor H89 treatment group before 4 hr hypoxia and 18 hr reperfusion injury.

We also performed RT-PCR (Figure 4) to assess MAP2, a protein essential to neurite growth [41, 42]. The mRNA level of MAP2 in H/R injured N2A cells was reduced considerably compared to the control group (Figure 4). We conclude that H/R injury leads to reduction of neurite outgrowth, which can be alleviated by H89 pretreatment. Thus, H89 may ameliorate the effects of H/R injury.

### 3.3. Cell Survival Was Increased in H89 Pretreated Neuro2A Cells after H/R Injury

**Figure 5:**
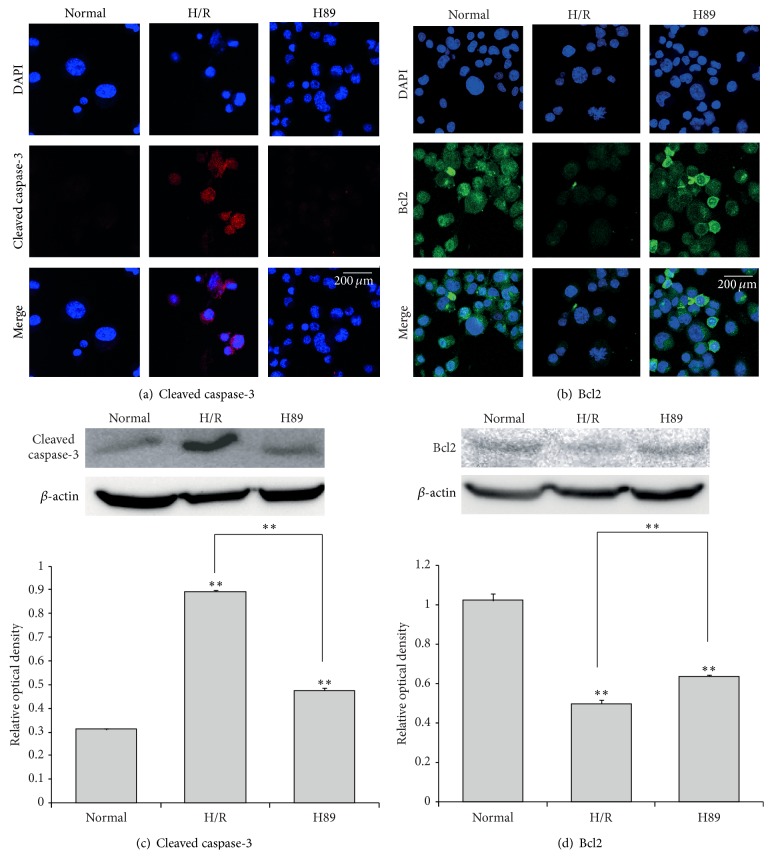
The measurement of cleaved caspase-3 and Bcl2 expression in Neuro2A cells after H/R-induced injury. (a) The level of cleaved caspase-3 was evaluated by immunocytochemistry. This image shows that the expression of cleaved caspase-3 in the H/R group was strongly increased compared to the normal group. Cleaved caspase-3 expression was attenuated in H89 pretreatment treatment group under H/R-induced injury. (b) The level of Bcl2 was evaluated by immunocytochemistry. This image shows that the expression of Bcl2 in the H/R group was increased compared to the normal group. PKA inhibitor H89 pretreatment preserved the expression of Bcl2 in spite of hypoxia reperfusion injury. (c) Western blotting experiments showed that the relative protein expression of cleaved caspase-3 evidently attenuated in the H89 group compared to the hypoxia reperfusion group. (d) Western blotting experiments showed that the relative protein expression of Bcl2 slightly increased in the H89 group compared to the hypoxia reperfusion group. Data were expressed as mean ± S.E.M, and each experiment included 4 repeats per condition. Differences were considered significant ^∗∗^ *P* < 0.001. Scale bar: 200 *μ*m, cleaved caspase-3: red, Bcl2: green, 4′,6-diamidino-2-phenylindole (DAPI): blue, normal: the normal control group, H/R: 4 hr hypoxia and 18 hr reperfusion injury group, and H89: 2 hr PKA inhibitor H89 treatment group before 4 hr hypoxia and 18 hr reperfusion injury.

To confirm whether or not H89 is involved in the neuronal cell death during H/R injury, we conducted the immunocytochemistry (Figures 5(a) and 5(b)), western blot analysis (Figures 5(c) and 5(d)), and RT-PCR (Figure 7(b)) using cleaved caspase-3 (as a marker of mitochondrial cell death) and Bcl2 (as a marker of anti-apoptosis) antibodies. H/R injured N2A cells were observed: the reduced Bcl2 immunoreactivity (Figure 5(b)), the decreased Bcl2 mRNA level (Figure 7(b)), the attenuated Bcl2 protein level (Figure 5(d)), the increased cleaved caspase-3 immunoreactivity (Figure 5(a)), and the increased cleaved caspase-3 protein level (Figure 5(c)). H89 pretreatment before H/R injury group showed the increased Bcl2 expression (Figures 5(b), 5(d), and 7(b)) and the reduced cleaved caspase-3 expression (Figures 5(a) and 5(c)) compared with the H/R group. These results indicated that the cell death in N2A cells was attenuated by H89 pretreatment in spite of hypoxia and reperfusion injury. Thus, we suggest that H89 may contribute to the neuronal cell survival pathway under hypoxia and reperfusion injury.

### 3.4. The Increase of BDNF Expression in Neuro2A Cells Pretreated with H89 in Hypoxia Reperfusion Injury

**Figure 6:**
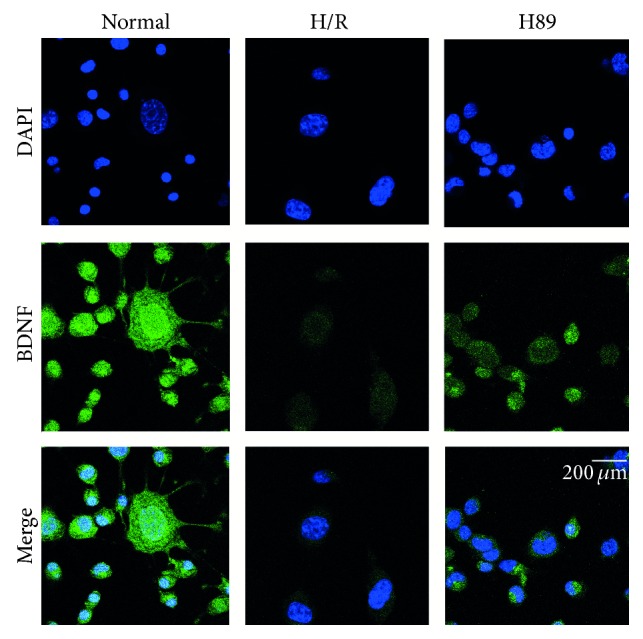
The measurement of BDNF expression in Neuro2A cells after H/R-induced injury. The level of BDNF was evaluated by immunocytochemistry. This image shows that the expression of BDNF in the H/R group was reduced compared to the normal group. H89 pretreatment increased the expression of BDNF in N2A cells in spite of hypoxia reperfusion injury. Scale bar: 200 *μ*m, BDNF: green, 4′,6-diamidino-2-phenylindole (DAPI): blue, normal: the normal control group, H/R: 4 hr hypoxia and 18 hr reperfusion injury group, and H89: 2 hr PKA inhibitor H89 treatment group before 4 hr hypoxia and 18 hr reperfusion injury.

**Figure 7:**
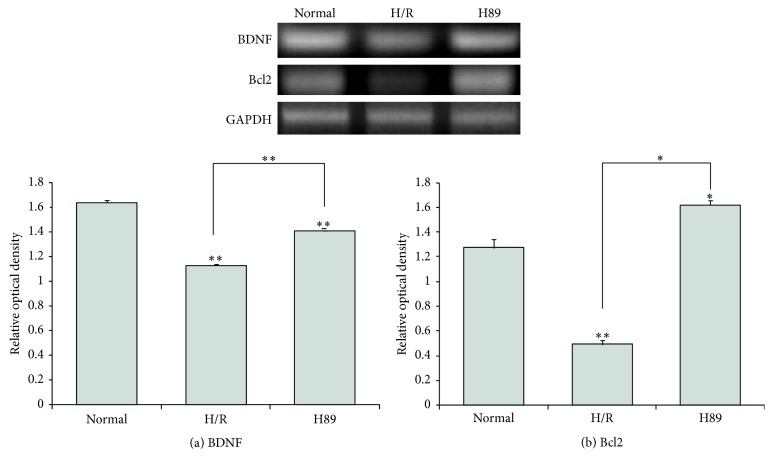
The measurement of BDNF and Bcl2 mRNA level in Neuro2A cells after H/R-induced injury. (a) BDNF and (b) Bcl2 mRNA levels were measured by using RT-PCR. The H89 pretreatment group showed higher mRNA levels of (a) BDNF and (b) Bcl2 compared to the hypoxia reperfusion injury group. Data were expressed as mean ± S.E.M, and each experiment included 3 repeats per condition. GAPDH was used as a control. Differences were considered significant at ^∗^ *P* < 0.05 and ^∗∗^ *P* < 0.001. Normal: the normal control group, H/R: 4 hr hypoxia and 18 hr reperfusion injury group, H89: 2 hr PKA inhibitor H89 treatment group before 4 hr hypoxia and 18 hr reperfusion injury.

We performed immunocytochemistry analysis (Figure 6) and RT-PCR (Figure 7(a)) using BDNF as the representative of neurotrophic factors in N2A cells to examine whether there was the alteration of neurotrophic factor expression in H89 pretreated N2A cells under hypoxia and reperfusion injury. We observed evidently lesser immunoreactivity of BDNF (Figure 6) in the H/R injured N2A cells compared to the normal control group. However, BDNF- (Figure 6) positive cells were obviously more expressed in H89 pretreated N2A cells than the H/R injury group. In addition, the BDNF mRNA level in N2A cells was higher in H89 pretreated N2A cells than the H/R injury group. These results showed that hypoxia and reperfusion stress suppresses the expression of BDNF in N2A cells, whereas H89 pretreatment H/R injured N2A cells considerably did not reduced the expression of BDNF against H/R injury. Based on these consequences, our results suggest that neurotrophic factor BDNF's expression was not reduced by H89 pretreatment despite ischemic injury. Thus, H89 may contribute to the expression of BDNF in N2A cells following hypoxia and reperfusion stress.

### 3.5. The Preservation of PSD-95 Expression in Neuro2A Cells Pretreated with H89 during Hypoxia Reperfusion Injury

**Figure 8:**
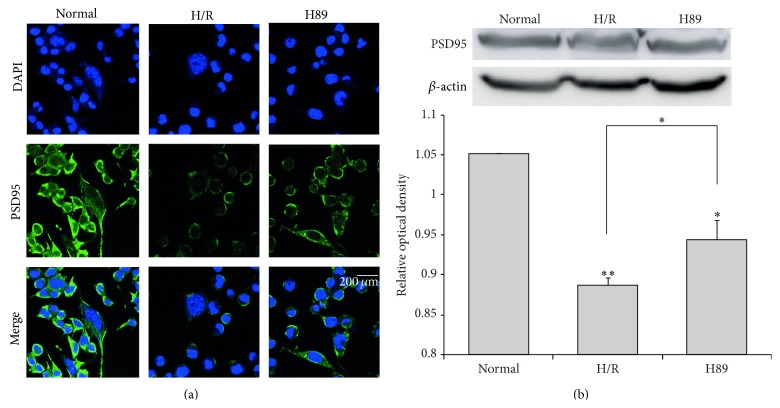
The measurement of PSD-95 expression in Neuro2A cells after H/R-induced injury. The level of PSD-95 was evaluated by immunocytochemistry. This image shows that the expression of PSD-95 in the H/R group was decreased compared to the normal group. PKA inhibitor H89 pretreatment increased the expression of PSD-95 in spite of hypoxia reperfusion injury. (b) Western blotting experiments showed that the relative protein expression of PSD-95 slightly increased in the H89 group compared to the hypoxia reperfusion group. *β*-actin was used as an internal control. Data were expressed as mean ± S.E.M, and each experiment included 4 repeats per condition. Differences were considered significant at ^∗^ *P* < 0.05 and ^∗∗^ *P* < 0.001. Scale bar: 200 *μ*m, PSD-95: green, 4′, 6-diamidino-2-phenylindole (DAPI): blue, normal: the normal control group, H/R: 4 hr hypoxia and 18 hr reperfusion injury group, and H89: 2 hr PKA inhibitor H89 treatment group before 4 hr hypoxia and 18 hr reperfusion injury.

We performed immunocytochemistry analysis (Figure 8(a)) and western blot analysis (Figure 8(b)) using PSD-95 antibody in N2A cells to investigate whether there was the alteration of synaptic plasticity related proteins in H89 pretreated N2A cells under hypoxia and reperfusion injury. In addition, we confirmed evidently decreased immunoreactivity of PSD-95 (Figure 8(a)) in the H/R injured N2A cells compared to the normal control group. On the other hand, the immunoreactivity of PSD-95 was more increased in H89 pretreated N2A cells than the H/R injury group (Figure 8(a)). Moreover, the protein level of PSD-95 (Figure 8(b)) in N2A cells was slightly higher in H89 pretreated N2A cells than the H/R injury group. These results indicated that hypoxia and reperfusion stress reduced the expression of PSD-95 in N2A cells, whereas H89 pretreatment H/R injured N2A cells considerably did not reduce expression of PSD-95 against H/R injury compared to H/R injured N2A cells. It is possible to extrapolate these results to suggest that the H/R injury reduced the expression of PSD-95. Data tend to support the conclusion that H89 may alleviate the synaptic plasticity damage of N2A cells against ischemic stress.

### 3.6. The Measurement of Phosphorylation AKT Protein Level in H89 Pretreated Neuro2A Cells against Hypoxia Reperfusion Injury

**Figure 9:**
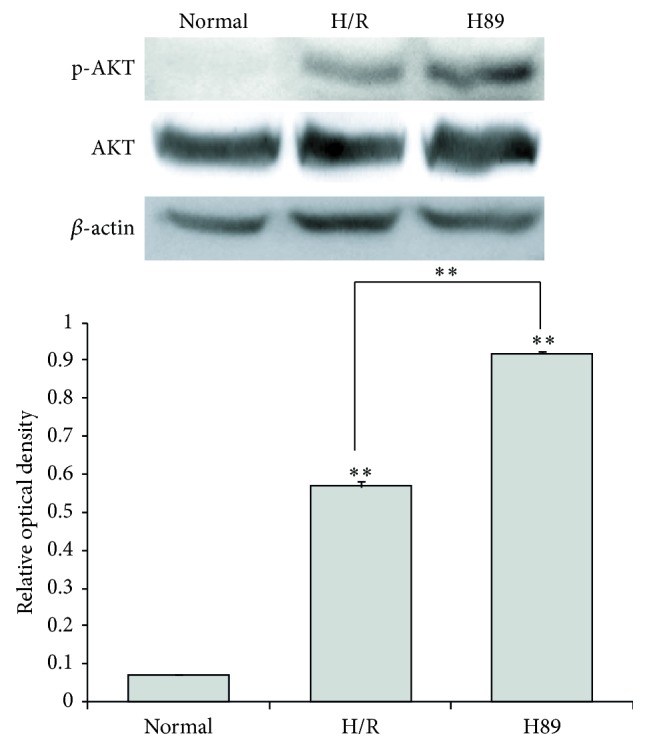
The measurement of AKT phosphorylation in Neuro2A cell against H/R injury. Western blotting experiments showed that the relative protein level of phosphorylation-AKT (p-AKT)/AKT significantly was increased in the H89 pretreatment group compared to the hypoxia and reperfusion group. *β*-actin was used as an internal control. Data were expressed as mean ± S.E.M, and each experiment included 4 repeats per condition. Differences were considered significant at ^∗∗^ *P* < 0.001. Normal: the normal control group, H/R: 4 hr hypoxia and 18 hr reperfusion injury group, H89: 2 hr PKA inhibitor H89 treatment group before 4 hr hypoxia and 18 hr reperfusion injury, and p-AKT: phosphorylation-AKT.

We performed western blot analysis (Figure 9) using AKT and phosphorylation-AKT (p-AKT) antibody in N2A cells to investigate the change of AKT phosphorylation in H89 pretreated N2A cells under hypoxia and reperfusion injury. The protein level of phosphorylation-AKT (Figure 9) was evidently increased in H89 pretreated H/R injured N2A cells than the H/R injury group. This result shows that H89 considerably promotes the activation of AKT signaling in N2A cells against H/R injury. Our data supports the hypothesis that H89 may boost the phosphorylation of AKT in N2A cells to survive the cells against ischemic stress.

## 4. Discussion

In cerebral ischemia, the reduction of synaptic dysfunction and neuronal cell loss are important issues and are implicated in severe pathogenesis such as memory impairment following ischemic stroke [26–29, 52, 53]. In the search for a solution, many researchers study the molecules and the signal pathways that lead to reduced synaptic plasticity and cell death [54–56]; an example of one is PKA signaling [3–7]. H89, known as the molecule commonly used to inhibit PKA action, recently has been reported to have a variety of functions unrelated to its effect on PKA inhibition [16, 18, 21, 57]. H89 affects ROCK II and, through that effect, cell morphology [48] and neurite extension [58, 59]. The data presented here indicate that H89 promotes neurite outgrowth and protects it after hypoxia stress. MAP2 (known as the neuron specific cytoskeletal protein) is present during all stages of neuromorphogenesis [60] and is necessary for neurite initiation [60–62]. Our MAP2 expression data support the contention that H89 may also support neurite outgrowth through MAP2. We speculate that the maintenance of neurite outgrowth after ischemic stroke is central to the role of H89. Several studies have demonstrated that H89 induces autophagy in cells independent of PKA signaling [24, 25] and increases cell survival after inflammation [54, 63]. In the present study, we observed reduced expression of cleaved caspase-3 and increased expression of Bcl2 following pretreatment with H89, supporting the conclusion that H89 protects against hypoxia injury, specifically, that it increases neuronal cell survival rate after ischemic stroke. Neurotrophic molecules regulate synaptic plasticity of the nervous system [64–66]. Specifically, many researches demonstrated that BDNF accelerates the axogenesis [30–32], promotes poststroke plasticity in an in vivo study [32, 67–71], and contributes to healthy brain function, notably, neuronal survival and maintenance, neurogenesis, modulation of dendritic branching and dendritic spine morphology [72, 73], and development of neuronal connections required for learning and memory [74–76]. BDNF, through phosphorylation of its TrkB receptor, activates a neuron-specific protein, controls the actin cytoskeleton in dendritic spines [77] and their regression [78, 79], and promotes the actin polymerization [80]. Inhibition of BDNF synthesis results in smaller spine heads and impairs long-term potentiation of synaptic transmission [81, 82]. Moreover, BDNF signaling plays a crucial role in the development of synapses by controlling the transport of PSD-95, which is the major scaffolding protein at mature glutamate synapses [83, 84]. PSD-95 itself and its interaction with BDNF signaling have been implicated in diverse brain diseases [85–87]. When localized in postsynaptic terminals, PSD-95 has an important role in postsynaptic function and plasticity [88–90]. The loss of PSD-95 results in severe cognitive decline due to loss of neurons and synaptic disruption [91–93]. In addition, synaptophysin as a marker of the pre-synaptic nerve terminal density is essential for vesicle fusion and the release of neurotransmitter [94]. The reduction of synaptophysin has been reported to reduce synaptic plasticity in the brain [95, 96]. Our results suggest that H89 may enhance synaptic plasticity by promoting the BDNF expression in neuronal cells under ischemic brain injury. Also H89 may be involved in neurite outgrowth by regulating the preservation of synaptic proteins, such as PSD-95 and synaptophysin, following ischemic brain damage. AKT which is activated by phosphatidylinositol 3-kinase activity [97] has known to promote a cellular protection after ischemic injury in the brain [98]. Moreover, AKT has been reported that it mediates anti-apoptosis signalings in ischemic stroke studies [99, 100]. Some study indicated that H89 markedly enhances the phosphorylation of AKT [101]. Considering our results, we assume that H89 may contribute to the survival of neuronal cells against ischemic injury through the activation of AKT. In present study, although learning and memory were not assessed in the animal model used here and we has some limitations to identify the specific molecular mechanism by H89, we propose that H89 may ameliorate the pathophysiology following ischemic stroke by reducing neuronal cell death and involving synaptic plasticity.
